# TCRP1 activated by mutant p53 promotes NSCLC proliferation via inhibiting FOXO3a

**DOI:** 10.1038/s41389-022-00392-9

**Published:** 2022-04-22

**Authors:** Hao Liu, Xiaoting Jia, Kai Luo, Xiangzhou Chen, Zhijie Zhang, Danyang Chen, Yixue Gu, Zhimin He, Guopei Zheng

**Affiliations:** Affiliated Cancer Hospital & Institute of Guangzhou Medical University, Guangzhou Key Laboratory of “Translational Medicine on Malignant Tumor Treatment”, Hengzhigang Road 78#, Guangzhou, 510095 Guangdong China

**Keywords:** Non-small-cell lung cancer, Ubiquitylation, Cell growth, Mutation

## Abstract

Previously, our lab explored that tongue cancer resistance-associated protein (TCRP1) plays a central role in cancer chemo-resistance and progression. Absolutely, TCRP1 was significantly increased in lung cancer. But the mechanism is far from elucidated. Here, we found that TCRP1 was increased in p53-mutant non-small-cell lung cancer (NSCLC), comparing to that in NSCLC with wild type p53. Further study showed that mutant p53 couldn’t bind to the promoter of TCRP1 to inhibit its expression. While the wild type p53 did so. Next, loss-and gain-of-function assays demonstrated that TCRP1 promoted cell proliferation and tumor growth in NSCLC. Regarding the mechanism, TCRP1 encouraged AKT phosphorylation and blocked FOXO3a nuclear localization through favoring FOXO3a ubiquitination in cytoplasm, thus, promoted cell cycle progression. Conclusionly, TCRP1 was upregulated in NSCLC cells with mutant p53. TCRP1 promoted NSCLC progression via regulating cell cycle.

## Introduction

International Agency for Research on Cancer (IARC) estimated that 19.3 million new cancer cases and almost 10.0 million cancer deaths occurred in 2020 [1]. Although lung cancer, with an incidence accounts for 11.4% in all cancers, is the second most common cancer diagnosed. It remained the leading cause of cancer death, with an estimated 1.8 million deaths (almost 18% in all cancers) [1]. The average 5-year survival of lung cancer (16.8%) is among the poorest of all cancers [2]. Non-small-cell lung cancer (NSCLC) comprises 85% of all lung cancers and has an average 5-year survival rate of 4% in cases of metastatic disease [3]. Therefore, it is of great value to reduce morbidity and mortality of NSCLC.

Current treatment for NSCLC includes surgical therapy, chemotherapy, and radiotherapy, but these treatments only modestly improve the survival of NSCLC patients [4]. Multiple attempts have been made to obtain a driver gene expression signature of NSCLC. Many genes, including epithelial growth factor receptor (EGFR), echinoderm microtubule-associated like 4-anaplastic lymphoma kinase (EML4-ALK), kirsten rat sarcoma viral oncogene homolog (KRAS), b-raf proto-oncogene serine/threonine-protein kinase (BRAF) and so on, were explored as functional driver genes in lung cancer [5, 6]. These genes aberrantly activated a series of downstream related signal pathways via mutation, amplification, or inactivation to modulate cell proliferation and survival in NSCLC [7–10]. Dramatic clinical response to epidermal growth factor receptor-tyrosine kinase inhibitors (EGFR-TKIs) demonstrated that these inhibitors triggered massive tumor apoptosis in NSCLC with EGFR mutation [11]. However, the effect of these inhibitors on NSCLC with EGFR mutation almost lasted only 10 to 13 months [12]. It is reported that KRASG12C inhibitor sotorasib elicited responses in about a third of NSCLC patients but soon it was generally well tolerated [13]. Thus, novel driver genes illustrating the lung pathogenesis and new targets for clinical treatment were warranted to improve the prognosis of NSCLC patients.

Previously, our lab analyzed the different expression genes in tongue cancer drug-resistant cell line Tca8113/PYM [14] and its parent cell Tca8113. A new expressed sequence tag (EST) attracted our attention. Subsequently, we identified a new gene (GeneBank ID: EF363480) and named it as *tongue cancer resistance-associated protein (TCRP1)* [15]. *TCRP1*, locates on human chromosome 11q13.4, produces an mRNA of 1834 nucleotides and translated into a protein having 235 amino acids [15]. Briefly, we investigated the chemoresistance of TCRP1 in tongue cancer and lung cancer cells. It is elucidated that TCRP1 mediated a specific resistance to cisplatin in Tca8113 cells through decreasing the cisplatin-induced apoptosis [15]. A large amount of studies have demonstrated that TCRP1 accelerated the activity of PI3K/Akt/NF-κB signal pathway to decrease the apoptosis of oral squamous cell carcinoma (OSCC) cells and to improve the survival of these patients [16–18]. The further study found that TCRP1 was contributed to cisplatin (cDDP) resistance in lung cancer cells through the prevention of Pol β degradation [19]. Additionally, TCRP1 was associated with resistance to cDDP and L-OHP via enhancing Akt/NF-κB signaling pathway in lung and ovarian cancer cells [20]. Coincidentally, it is noted that TCRP1 induced tamoxifen resistance in breast cancer cells. TCRP1 promoted SGK1 activation via phosphorylation of PDK1 in breast cancer cells [21]. Recently, we found that TCRP1 was often upregulated in human cancer tissues, such as lung cancer, glioma, ovarian cancer, thyroid cancer, nasopharyngeal carcinoma, pancreatic cancer, stomach cancer, tongue carcinoma, and chronic myeloid leukemia (CML) [22, 23]. However, the mechanism of TCRP1 in NSCLC remained incompletely clarified.

Here, we determined TCRP1 expression pattern in NSCLC, and found mutant p53 could evidently enhance the expression of TCRP1 in NSCLC. TCRP1 was positively correlated with poor prognosis of NSCLC patients. In addition, we also discovered that TCRP1 promoted FOXO3a degradation to promote the cell proliferation and tumor growth of NSCLC.

## Material and methods

### Cells and specimens

Lung cancer cell lines (A549, H1299, H1975, HCC827, and H460) were cultured in 1640 (Gibco, USA) supplemented with 10% fetal bovine serum (Gibco, USA). All these cells were incubated at 37 °C with 5% CO_2_ in a humidified incubator. All the cell lines were reauthenticated by short tandem repeat analysis every 6 months after resuscitation in our laboratory.

41 cases of normal tissues and 133 cases of NSCLC tissues were collected from patients in the Affiliated Cancer Hospital & Institute of Guangzhou Medical University between May 2012 and July 2019. All the clinical data, such as age, tumor size, TNM stage, lymph node status, and distant metastasis were obtained from clinical and pathologic records. Among these NSCLC tissues, there were 90 cases including patients’ prognosis information. Overall survival was calculated from the day of surgery to the day of death or of the last follow-up. Another cohort of 74 cases NSCLC tissues were obtained from the biological resource specimen bank of Affiliated Cancer Hospital & Institute of Guangzhou Medical University. Written informed consent was provided by all patients based on the Declaration of Helsinki. These studies were approved by the ethics committee of Affiliated Cancer Hospital & Institute of Guangzhou Medical University.

### Bioinformatics analysis

We obtained the TCRP1 expression and p53 mutation information from The Cancer Genome Atlas Project (TCGA) databank (https://portal.gdc.cancer.gov/). Promoter sequence of TCRP1 were downloaded from UCSC (http://genome.ucsc.edu/), and potential transcriptional factors were predicted via JASPAR (http://jaspardev.genereg.net/).

### Plasmids construction

CRISPR-CAS9 plasmids for knock-down TCRP1, overexpression plasmids for TCRP1 and FOXO3a, plasmids for shRNAs targeting FOXO3a and p53 were bought from GeneChem biotechnology Limited (China). Overexpression plasmids for wildtype p53, p53R175H, p53V143A and the control group were kept in our lab.

Total DNA were extracted from A549 cells as template, primers were listed in Table 1. The fragment of wildtype promoter of TCRP1 were got via PCR assays. The pGL4 Basic vector and PCR fragments were digested by *Xho* I and *Hind* III restriction endonuclease, constructed by T4 DNA ligase, and sequenced. The positive clone were named pGL4-wt. The mutant reporter plasmids were constructed by MutaBEST Kit (TaKaRa, Japan) following users’ instruction, and the target clone were named pGL4-mut-1, pGL4-mut-2, and pGL4-mut-all.

**Table 1:**
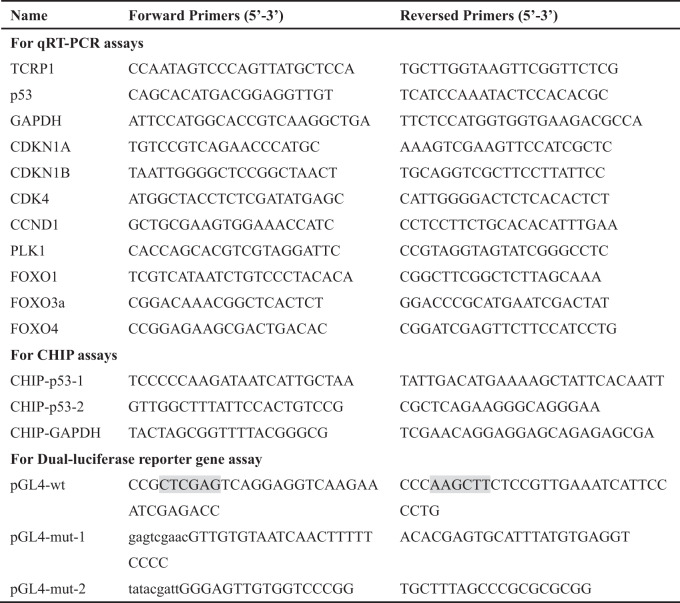
Summary of primers in this study.

The shaded area is the restriction endonuclease site, and the lowercase letters indicate the p53 mutation site.

### RNA extraction and qRT-PCR

Total RNAs were extracted by TRIzol Reagent (Thermo Fisher Scientific, USA), and then were reversely transcribed into cDNA via RevertAid First Strand cDNA Synthesis Kit (Thermo Fisher Scientific, USA). qRT-PCR was performed using SYBR-Green PCR Master Mix (Thermo Fisher Scientific, USA). Specific primers have been listed in Table 1.

### Western blot

Cells were lysed via RIPA buffer (Thermo Fisher Scientific, USA) with Cocktail protease inhibitor (Thermo Fisher Scientific, USA). The protein concentration was determined using BCA Protein Assay Kit (Thermo Fisher Scientific, USA). Equal amounts of protein were loaded in each SDS-PAGE lane, electrophoresed, and transferred onto PVDF membranes. The membranes were blocked with 5% non-fat dry milk, incubated with primary antibodies, and followed by HRP-conjugated secondary antibody. The signal was detected using ECL detection system (Millipore, USA) as described by the manufacturer. The antibodies were used as below: TCRP1 antibody (abcam, #ab254815), FOXO1 antibody (CST, #2880), FOXO4 antibody (CST, #2499), FOXO3a antibody (CST, #12829), phospho-FOXO3a (Thr 32) antibody (Sigma, ABS554), AKT antibody (CST, #4691), phospho-Akt (Ser473) antibody (CST, #4060), p21 antibody (CST, #2947), p27 antibody (CST, #3686).

### Dual-luciferase report gene assay

Cells were seeded into 96-well plates at a concentration of 1 × 10^5^ cells per well overnight. 0.3 μg Overexpression plasmids of wt-p53, p53R175H and p53V143A were co-transfected with 0.2 μg reporter plasmid (pGL4-wt, pGL4-mut-1, pGL4-mut-2, or pGL4-mut-all) and 0.02 μg pRLTK (a negative control) into a well, respectively. 48 h later, cells were incubated with passive lysis buffer for 30 mins. And then the luciferase activities of Firefly and Renilla were estimated by a BioTek Synergy 2 reader (BioTek Instruments, USA).

### Cell viability assay

30,000 target cells per well were seeded into 6-well plate. Each group cells were seeded into three wells for repeat. Cell numbers were counted at 0, 24, 48, 72, and 96 h. The cell viability curves were drawn basing on cell numbers.

### BrdU assay

A total of 10,000 cells per well were seeded into 96-well plates. BrdU solution, BrdU antibody, and HRP-conjugated secondary antibody was added into cells in turn following instruction. The absolute absorbance at 450 nm was recorded by a BioTek Synergy 2 reader. The absorbance represented the cell number, with the absorbance of the control set as 100%.

### Colony Formation Assay

For colony-formation assay, 500 cells were seeded per well in 6-well-plate. The cells were incubated at 37 °C with 5% CO_2_ in a humidified incubator. After 2 weeks, the cells were fixed in methanol and stained with 0.2% crystal violet. Number of colonies was counted.

### Chromatin Immunoprecipitation (ChIP)

ChIP assays were performed via SimpleChIP® Plus Enzymatic Chromatin IP Kit (Cell Signaling Technology, USA) according to the manufacturer’s protocol. At the beginning, cells were fixed with 4% formaldehyde, collected, and resuspended in lysis buffer. Cells were sonicated five times for 5 s at 30% of maximal power. Moreover, cells were centrifugated. The supernatant was equally separated into 3 groups. p53, IgG and polymerase II antibodies were added into each group. Further, these cell lysates with different antibodies were incubated with Protein G magnetic beads. The antibody/protein/nucleic acid complex were reversely cross-linked by heating at 65 °C, digested with proteinase K at 37 °C, treated with RNase A at 37 °C. DNA was obtained by phenol and phenol/chloroform extractions. PCR was implemented, and primers are listed in Table 1.

### Flow cytometry

Cell cycle was estimated by Cell Cycle Staining Kit (MultiSciences, China) following the instructor’s protocol. A total of 1 × 10^6^ cells were harvested, incubated with DNA Staining solution and Permeabilization solution. Mentioned cells were analyzed by flow cytometry (BD Company, USA).

### Immunohistochemistry (IHC) assay

Briefly, the sections were deparaffinized in xylene, rehydrated with graded alcohol, and then boiled in 0.01 M citrate buffer (pH 6.0) for 20 min with an autoclave. The sections were incubated in hydrogen peroxide (0.3%) to block endogenous peroxide activity, and then were added with normal goat serum to reduce nonspecific binding. Furthermore, these sections were incubated with the primary antibodies and the secondary antibody in turn. Additionally, sections were counterstained with haematoxylin and dehydrated. The scores of each section were multiplied to give a final score of 0–12, and the tumors were finally determined as negative (−), score 0; lower expression (+), score ≤ 4; moderate expression (++), score 5–8; and high expression (+++), score ≥ 9. All immunohistochemical staining were evaluated and scored by at least two independent pathologists.

### Immunofluorescence (IF) assay

Generally, cells grew on cover slips were fixed with 4% paraformaldehyde, and were permeabilized by 0.1% Triton X-100. Next, these cells were incubated with primary antibody for 1 h, and followed with secondary antibody. Also, these cells were incubated with DAPI to stain nucleus. Images were acquired on a confocal microscope (Zeiss, Germany).

### Co-immunoprecipitation (Co-IP) assay

Cells were dissolved with lysis buffer (Thermo Fisher Scientific, USA), ultrasonic disrupted, and centrifugated. The supernatant of cell lysate were divided into three group: 50 μL were used as Input. The rest was equally separated into 2 parts (One for IgG group, the other is for target antibody). Target antibody (anti-FOXO3a or anti-ubiquitin) and IgG were incubated with the cell lysate overnight, respectively. Protein A/G Magnetic Beads (Thermo Fisher Scientific, USA) were added into above mixture for 1 h. The protein/antibody/beads complex were separated by a magnetic stand. These samples were used for western blot.

### Animal experiments

4-week-old female nude mice were purchased from Guangdong Medical Laboratory Animal Center (Guangzhou, China) and were bred following the protocol of Guangdong Medical Laboratory Animal Center. All animal work was approved by the Animal Experimentation Ethics Committee of Affiliated Cancer Hospital & Institute of Guangzhou Medical University. The mice were randomly divided into groups. To evaluate the effect of TCRP1 on tumor growth, control cells (left) and TCRP1 KD cells (right) were subcutaneously injected into the nude mice. Tumor size and body weight were measured every 3 days. The tumor volume was calculated using the formula: V = 1/2 × larger diameter × (smaller diameter)^2^, and growth curves were plotted using average tumor volume within each experimental group at the set time points. The tumors were removed and weighed, finally were used in IHC assays.

### Statistical analysis

All the experiments were performed for at least three times, and data were represented as mean ± S.D. Unpaired Student’s t-test was used to assess differences between groups. High and low expression groups were defined as above and below the mean expression level, respectively. Kaplan-Meier survival curves were generated using Prism software and a log-rank test was performed to assess statistical significance between groups. *P* values < 0.05 were considered statistically significant.

## Results

### TCRP1 expression is highly elevated in p53-mutant NSCLC

**Fig. 1:**
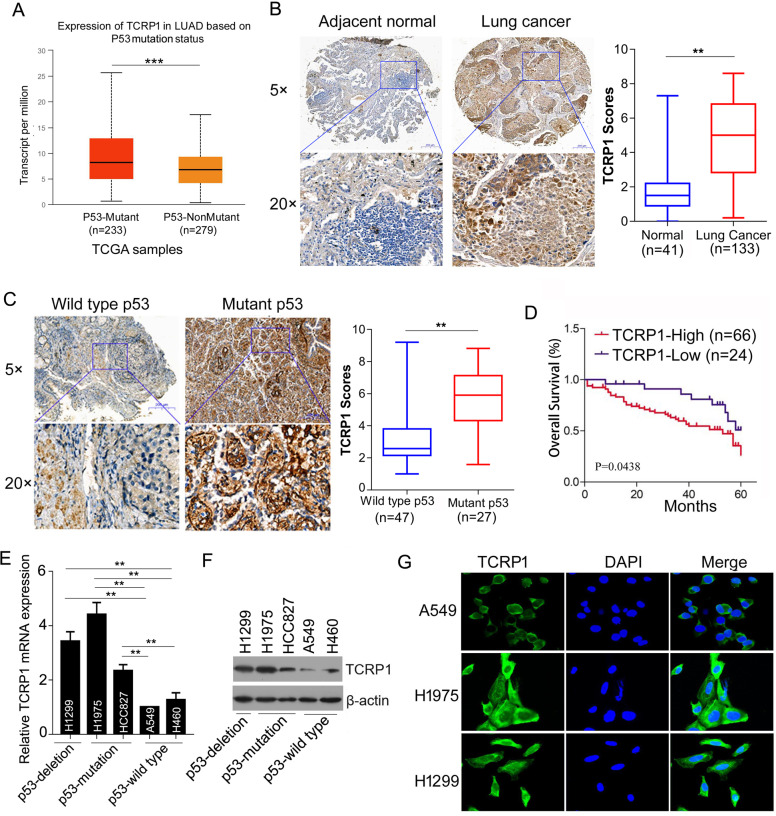
TCRP1 was enhanced in p53-mutant NSCLC. **A** TCRP1 expression in LUAD was measured basing on p53 mutation from TCGA databanks. *vs* p53-mutant, ^***^P < 0.001. **B** IHC assays were performed to evaluate TCRP1 expression in 41 cases of normal tissues and 133 cases of NSCLC tissues. *vs* Normal, ^**^P < 0.01. **C** TCRP1 expression in NSCLC tissues with wild type p53 and mutant p53 were determined via IHC assays, respectively. *vs* wild type p53, ^**^P < 0.01. **D** The overall survival of 90 NSCLC patients with high or low expression of TCRP1 were assessed by Kaplan-Meier plot analysis. **E** The mRNA expression of TCRP1 were detected in lung cancer cells by qRT-PCR assays. Among these cells, H1299 is p53 deletion, H1975 and HCC827 are p53 mutation, while A549 and H460 are p53 wild type. *vs* A549, *vs* H460, ^**^P < 0.01. **F** The protein expression of TCRP1 were assessed in lung cancer cells using western blot assays. **G** IF assays were conducted to estimate the expression of TCRP1 in NSCLC cells.

We previously identified that TCRP1 is highly expressed in lung cancer compared with normal tissue base on TissueScan™ Cancer and Normal Tissue cDNA Arrays [22]. To investigate the mechanism by which TCRP1 expression is highly elevated in lung cancer, we analyzed The Cancer Genome Atlas (TCGA) data sets to determine whether upregulated TCRP1 expression correlates with gene copy number, promoter methylation, or any specific oncogenic mutations. Our analysis revealed that enhanced TCRP1 expression is significantly enriched in NSCLC with p53 mutation in TCGA data sets (Fig. 1A). Next, we examined TCRP1 expression in 133 independent primary NSCLC tumor samples by immunohistochemistry analysis. The results showed that TCRP1 expression in NSCLC tissues is considerably higher than that in normal tissues (Fig. 1B). Importantly, p53-mutant NSCLC tumors expressed obviously elevated TCRP1 protein compared with the p53 wild-type tumors (Fig. 1C). Moreover, analysis of the correlation between TCRP1 expression and NSCLC patients’ overall survival showed that high TCRP1 expression is remarkably associated with poor survival in NSCLC patients (Fig. 1D). Furthermore, we found that TCRP1 mRNA and protein expression are elevated in p53-mutation and p53-deletion lung cancer cell lines compared with that in wild-type p53 cells (Fig. 1E–G). Collectively, TCRP1 was impressively accelerated in mutant p53 NSCLC than that in NSCLC with wild-type p53. And, TCRP1 was positively correlated with poor prognosis of NSCLC patients.

### Wild type p53 but not mutant p53 significantly inhibited TCRP1 expression

**Fig. 2:**
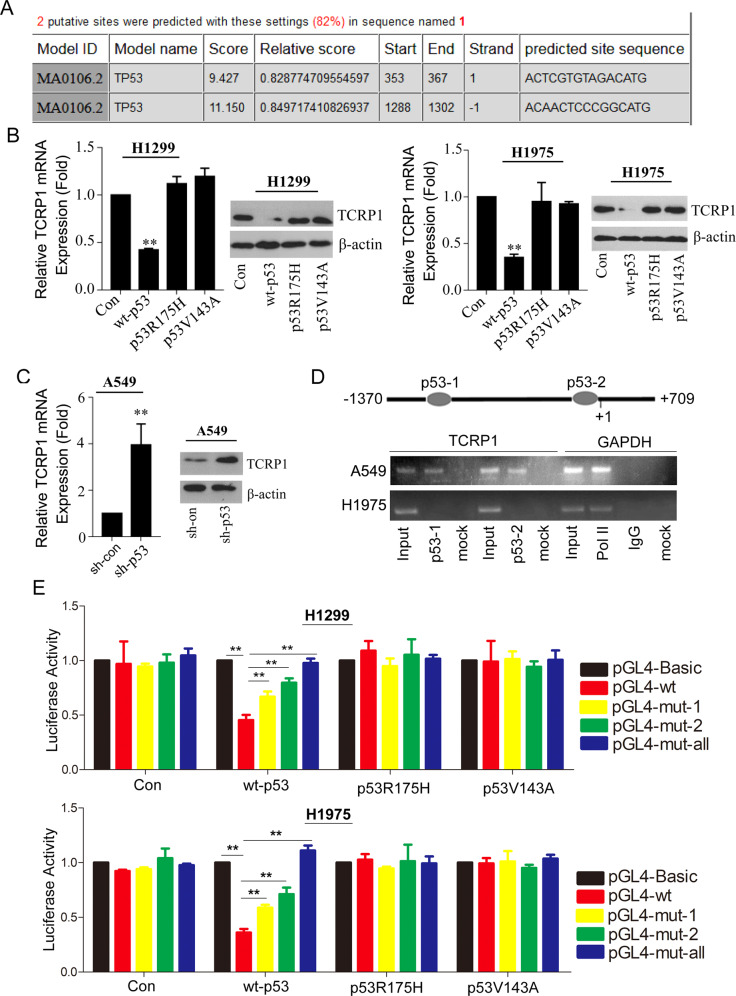
Mutant p53 enhanced TCRP1 in NSCLC. **A** Potential binding sites of transcriptional factor p53 in the promoter of TCRP1 were predicted via online software JASPAR. **B** Plasmids of wild type p53 (wt-p53) and mutant p53 (p53R175H and p53V143A) were transfected into H1299 and H1975 cells, the expression of TCRP1 in mentioned cells were estimated by qRT-PCR and western blot assays. *vs* Con, ^**^P < 0.01. **C** ShRNAs targeting p53 and control group were introduced into A549 cells, the expression of TCRP1 in these cells were estimated by qRT-PCR and western blot assays. *vs* sh-con, ^**^P < 0.01. **D** The schematic map of p53 binding sites on the promoter of TCRP1 were exhibited. CHIP assays were carried out to illustrate whether p53 could bind to the promoter of TCRP1. **E** Plasmids of pGL4-wt, pGL4-mut-1, pGL4-mut-2 and pGL4-mut-all were employed in H1299 and H1975 cells combing with wild type p53 (wt-p53) or mutant p53 (p53R175H and p53V143A), respectively. In above cells, dual-luciferase reporter assays were performed to measure the luciferase activity of each group. *vs* pGL4-Basic, *vs* pGL4-wt, ^**^P < 0.01.

The online software JASPAR indicated that there are two binding sites of transcriptional factor p53 in the promoter of TCRP1 (Fig. 2A). Previous reports have shown that gain-of-function p53 mutants induce the expression of several critical genes involved cancer initiation and progression. To determine whether mutant p53 modulated the expression of TCRP1, we generated stable cells to restore p53 mutants (R175H, V143A) in p53-deletion H1299 cells (Fig. S1A). The results demonstrated that overexpression of wild-type p53 significantly suppressed TCRP1 expression in both H1299 and H1975 cells. However, this effect would be reversed by overexpression of p53 mutants (Fig. 2B). Consistently, depletion of wild-type p53 using small interfering RNA (shRNA) significantly increased the expression of TCRP1 in A549 cells (Fig. S1B, Fig. 2C). Furthermore, CHIP assays suggested that wild-type p53 could bind to the TCRP1 promoter at both sites in A549 cells. While the mutant p53 in H1975 cells did not (Fig. 2D). Additionally, we conducted luciferase reporter assays in H1299 and H1975 cells with wild-type and mutant p53, respectively. The data demonstrated that wild-type p53 could significantly suppress the activity of wild-type TCRP1 promoter in H1299 and H1975 cells. The mutant p53 could change these consequences. Especially, either mutant binding site 1 or 2 could remarkably reverse the effect of wild-type p53 on activity of TCRP1 promoter (Fig. 2E). All together, wild-type p53 but not mutant p53 could suppress the expression of TCRP1.

### TCRP1 promotes NSCLC cell proliferation and tumor growth

**Fig. 3:**
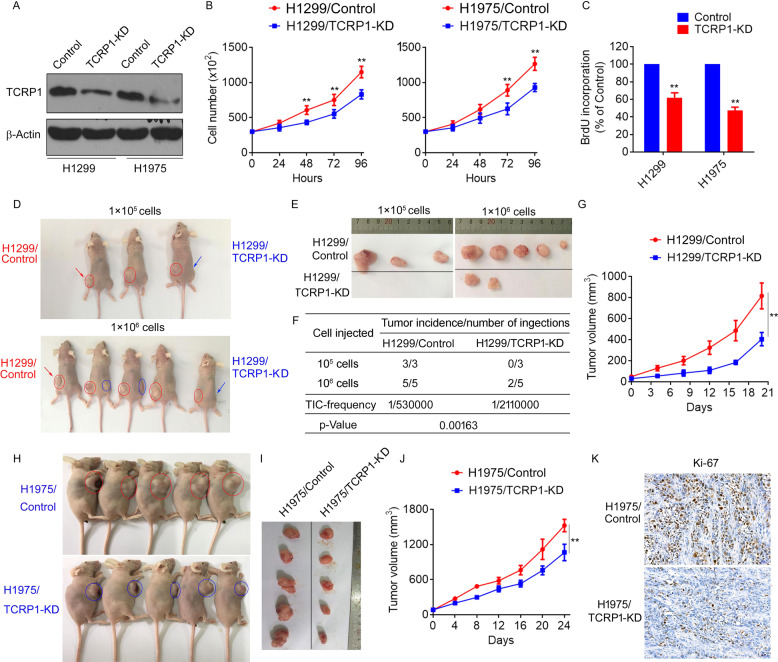
Knocking down TCRP1 suppressed NSCLC cell proliferation in vitro and in vivo. CRISPR-CAS9 technique was used to knock down TCRP1 in H1299 and H1975 cells. Protein expression of TCRP1 in mentioned cells was measured via western blot assay (**A**). The proliferation of above cells was evaluated by cell viability (**B**) and BrdU assays (**C**), *vs* Control, ^**^P < 0.01. (**D**) A total of 1 × 10^5^ and 1 × 10^6^ mentioned cells were subcutaneously injected into nude mice, respectively. The H1299/Control cells were injected at left, while the H1299/TCRP1 KD cells were injected at right. The tumors obtained from these mice were exhibited (**E**), the tumor incidence (**F**) and tumor growth curve were analyzed, *vs* Control, ^**^P < 0.01 (**G**). The H1975/Control cells were subcutaneously injected at left of nude mice, while the H1975/TCRP1 KD cells were at right. The tumors obtained from these mice were exhibited (**H**). The tumor growth curves were plotted using average tumor volume within each experimental group at the set time points (**I**). (**J**) IHC assays were performed to determine ki67 expression level in these tumors. *vs* Control, ^**^P < 0.01. (**K**) Ki-67 expression in tumors of H1975/Control and H1975/TCRP1 KD group were evaluated by IHC assays.

To explore the function of TCRP1 in NSCLC cells, we employed the CRISPR-CAS9 technique to knock down TCRP1 in H1299 and H1975 cells (Fig. 3A). We found that decreasing TCRP1 significantly reduced NSCLC cell viability (Fig. 3B). Similar results were obtained by BrdU methods (Fig. 3C). In clonogenic assays, the number of tumor cell colonies was evidently lower upon TCRP1-knockdown cells compared with control cells (Fig. S2A). Moreover, our results showed that experimentally overexpressing TCRP1 remarkably increased cell proliferation of A549 cells (Fig. S2B–D). To address the relevance of the above in vitro findings that TCRP1 promoted NSCLC cell proliferation, we established H1299 and H1975-xenograft tumors and evaluated the effect of TCRP1 knockdown on tumorigenicity and tumor growth. Two doses (1 × 10^6^, 1 × 10^5^) of TCRP1-knockdown H1299 cells or control cells were subcutaneously injected in nude mice, respectively. (Fig. 3D). Further, we found that TCRP1-knockdown H1299 cells showed a striking 4-fold reduction in tumor-initiating cell (TIC) frequency compared to control cells (Fig. 3E, F). Moreover, knockdown of TCRP1 in H1299 cells also significantly inhibited tumor growth (Fig. 3G). Consistently, TCRP1-knockdown H1975 cells displayed lower tumor growth rates than control cells (Fig. 3H–J). In addition, tumors formed by TCRP1-knockdown H1975 cells showed obviously downregulated levels of proliferative marker ki67 (Fig. 3K). These results indicated that TCRP1 significantly accelerates tumorigenicity and tumor growth in NSCLC.

### TCRP1 promotes NSCLC cell-cycle progression

**Fig. 4:**
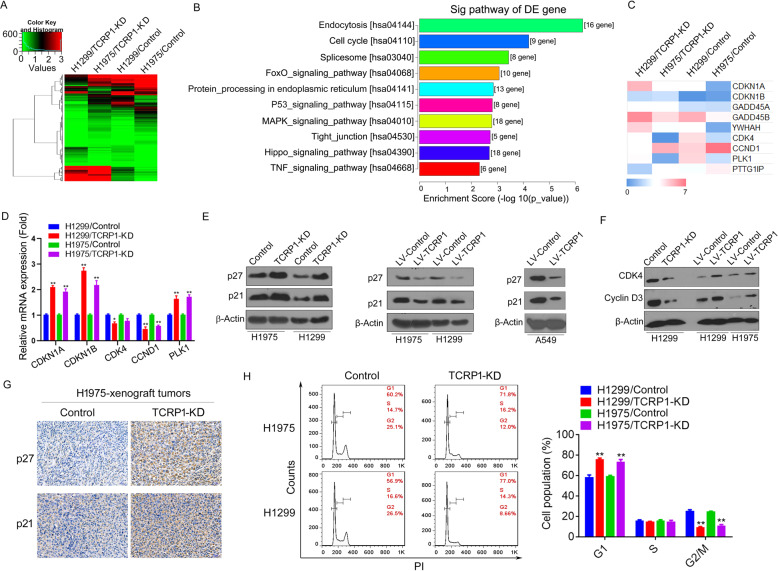
TCRP1 advances cell cycles of NSCLC cells. **A** Total RNAs were collected from TCRP1-knockdown H1299 and H1975 cells, as well as their control cells, respectively. RNA-sequencing assays were performed in mentioned groups. **B** The differently expressed (DE) genes in above groups were analyzed by GO-KEGG analysis. **C** The potential DE genes related to cell cycles were exhibited. **D** The whole RNAs from mentioned cells were obtained. The expression levels of target genes were estimated via qRT-PCR assays, respectively. vs Control, ^*^P < 0.05, ^**^P < 0.01. **E** TCRP1 was knocked down in H1299 and H1975 cells. Meanwhile, TCRP1 was overexpressed in H1299, H1975 and A549 cells. Western blot assays were performed to evaluate p21 and p27 protein expression in each group. **F** The protein levels of CKD4 and Cyclin D3 were determined via western blot assays in H1299 cells with increasing or decreasing TCPR1 and H1975 cells with enhancing TCRP1. **G** IHC assays were carried out to measure p21 and p27 levels of H1975 cells with TCRP1 knockdown and control groups in vivo. **H** TCRP1 was knocked down in H1299 and H1975 cells. The cell cycle of each group was detected via flow cytometry assays. *vs* Control, ^**^P < 0.01.

To further elucidate the mechanism by which TCRP1 modulating NSCLC progression, we performed RNA-sequencing assays from TCRP1-knockdown H1299 and H1975 cells (Fig. 4A). KEGG analysis revealed that the potential targets of TCRP1 were involved in cell cycle progression (Fig. 4B, C). The changes in the level of possible targets mediated by TCRP1 were confirmed via qRT-PCR assays (Fig. 4D). Western blot results further demonstrated that knockdown of TCRP1 significantly increased the protein expression levels of p27 and p21 in H1299 and H1975 cells (Fig. 4E). On the contrary, overexpression of TCRP1 notably reduced p27 and p21 expression in NSCLC cells (Fig. 4E). Furthermore, we found that knockdown of TCRP1 reduced Cyclin D3 and CDK4 expression, whereas overexpression of TCRP1 did the opposite (Fig. 4F). Subsequently, tumors formed by TCRP1-knockdown H1975 cells showed an evidently increased protein level of p27 and p21 (Fig. 4G). In addition, we examined whether the cell cycle was regulated by TCRP1 in NSCLC cells. Flow cytometry assays illustrated an obvious increase in the G1 phase population and a decrease in the G2/M phase population of TCRP1-knockdown H1299 and H1975 cells compared with the respective control groups (Fig. 4H). Thus, these results suggested that TCRP1 regulates cell cycle progression in NSCLC cells.

### TCRP1 inhibits FOXO3a expression in NSCLC

**Fig. 5:**
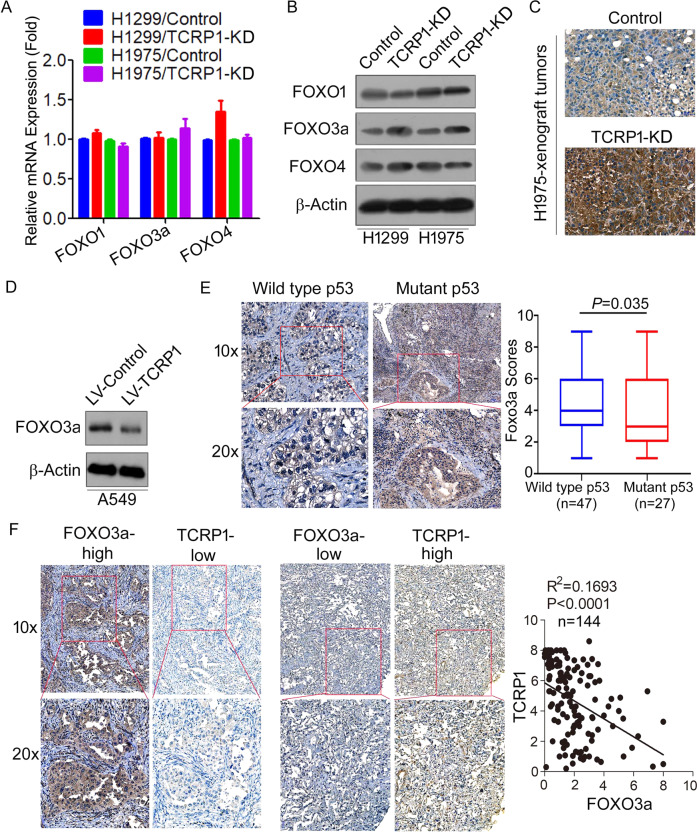
TCRP1 downregulated the protein level of FOXO3a. In H1299 cells and H1975 cells with knocking down TCRP1 and each control cells, qRT-PCR (**A**) and western blot assays (**B**) were utilized to detect the expression of FOXO transcription factors in these cells. **C** The levels of FOXO3a in tumors drove by H1975 cells with reducing TCRP1 and control groups were estimated by IHC assays. **D** The protein levels of FOXO3a in A549 cells with restoring TCRP1 and control cells were estimated by western blot assays. **E** IHC assays were implemented to evaluate FOXO3a expression in 74 cases of NSCLC tissues basing on wild type p53 or mutant p53. **F** The expression levels of TCRP1 and FOXO3a in 144 cases of NSCLC tissues were estimated via IHC assays. The expression relationship between TCRP1 and FOXO3a was analyzed by Pearson correlation analysis.

KEGG analysis revealed that the TCRP1-regulated genes were significantly enriched in FOXO signaling pathway (Fig. 4B). As the FOXO transcription factor plays an important role in the regulation of cell-cycle progression by transcriptionally regulating the expression of p21 and p27. We speculated that TCRP1 might promote NSCLC cell-cycle progression by repressing the FOXO transcription factor. Briefly, we found that the mRNA expression levels of FOXO transcription factor (FOXO1, FOXO3a, and FOXO4) were not obviously changed in TCRP1-knockdown cells compared to control cells (Fig. 5A). However, knockdown of TCRP1 significantly increased the protein expression levels of FOXO3a, but had no effect on FOXO1 and FOXO4 expression (Fig. 5B). Tumors formed by TCRP1-knockdown H1975 cells further were confirmed that knockdown of TCRP1 increased FOXO3a expression (Fig. 5C). On the contrary, overexpression of TCRP1 significantly reduced FOXO3a expression in A549 cells (Fig. 5D). We evaluated FOXO3a expression in 74 cases of NSCLC tissue samples by IHC assays. Importantly, 27 cases of p53-mutant NSCLC tumors expressed significantly decreased FOXO3a protein compared with 47 cases of wild type p53 tumors (Fig. 5E). Markedly, FOXO3a protein expression showed a highly significant (*P* < 0.0001) negative correlation with TCRP1 in 144 cases of NSCLC tissues (Fig. 5F). Collectively, our data provided an evidence for the physiologic relevance of TCRP1-mediated FOXO3a regulation in NSCLC.

### TCRP1 modulates AKT-mediated phosphorylation and nuclear localization of FOXO3a

**Fig. 6:**
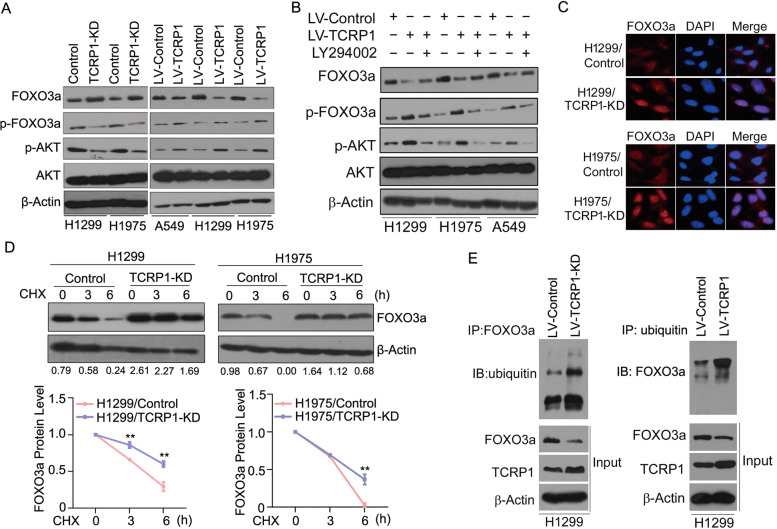
TCRP1 mediated nuclear location and expression of FOXO3a via AKT. In NSCLC cells with knocking down or increasing TCRP1 (**A**), and in NSCLC cells with overexpression of TCRP1 combing with PI3K inhibitor LY294002 (**B**), western blot assays were performed to measure the phosphorylation levels and protein levels of FOXO3a and AKT. **C** The nuclear localization of FOXO3a was exhibited via IF assays in H1299 and H1975 cells with decreasing TCRP1. (**D**) TCRP1-knockdown H1299 and H1975 cells were treated with CHX for 0, 3, and 6 h, western blot assays and Image J software were used to determine FOXO3a expression. *vs* Control, ^**^P < 0.01. (**E**) CO-IP assays were carried out to test the ubiquitination level of FOXO3a in H1299 cells with enhancing TCRP1.

We previously demonstrated that TCRP1 promotes cancer development by activation of AKT signaling. FOXO3a is a key target of AKT, and AKT phosphorylates FOXO3a at Thr32 residues, resulting in a decreased expression of FOXO3a. We then investigated whether TCRP1 inhibited FOXO3a expression via AKT-dependent manner. It is of note that knockdown of TCRP1 reduced phosphorylation levels of AKT at Ser473 in H1299 and H1975 cells. Consistently, the levels of FOXO3a phosphorylation at Thr32 were reduced in TCRP1-knockdown H1299 and H1975 cells (Fig. 6A). In contrast, overexpression of TCRP1 significantly increased the phosphorylation levels of FOXO3a and AKT, respectively (Fig. 6B). We further tested the role of AKT phosphorylating FOXO3a in a TCRP1-mediated manner. Consistent with AKT inhibition, treatment of PI3K inhibitor LY294002 markedly decreased phosphorylation levels of FOXO3a but increased protein levels of FOXO3a in TCRP1-overexpressing NSCLC cells (Fig. 6B). Phosphorylation of FOXO3a is a key event that determines its subcellular location and transcriptional activity. We then investigated whether TCRP1 takes part in the distribution of FOXO3a in NSCLC cells. Immunofluorescence analyses showed an increased nuclear localization of FOXO3a in TCRP1-knockdown H1299 and H1975 cells as compared with control cells (Fig. 6C). Given that the AKT-mediated phosphorylation promotes FOXO3a nuclear exclusion followed by degradation through the ubiquitin-proteasome pathway, we examined the stability of FOXO3a in TCRP1-knockdown H1299 and H1975 cells with cycloheximide (CHX). The results suggested that reducing TCRP1 evidently enhanced the stability of FOXO3a comparing with the control group (Fig. 6D). Further, we used co-immunoprecipitation (CO-IP) assay to evaluate the effect of TCRP1 on FOXO3a ubiquitination. The data showed that overexpression of TCRP1 significantly increased FOXO3a ubiquitination (Fig. 6E). These results suggested that TCRP1 inhibited the nuclear localization and expression of FOXO3a via AKT-mediated phosphorylation.

### TCRP1 promotes NSCLC cell-cycle progression by repressing the expression of FOXO3a

**Fig. 7:**
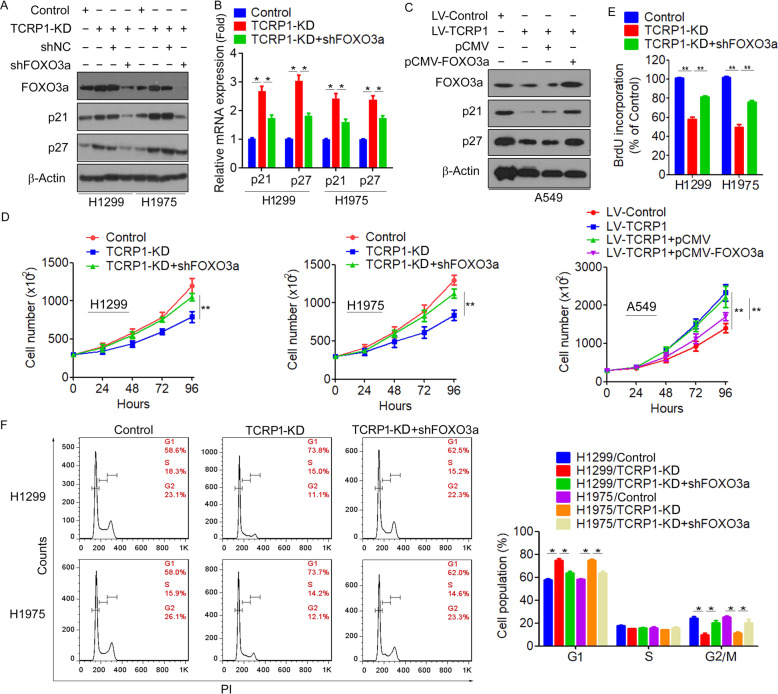
FOXO3a reversed the effect of TCRP1 on NSCLC cell proliferation. shRNA targeting FOXO3a was transfected into H1299 cells and H1975 cells with decreasing TCRP1, western blot (**A**) and qRT-PCR assays (**B**) were performed to assess the expression level of FOXO3a, p21 and p27. *vs* TCRP1-KD, *vs* Control, ^*^P < 0.01. (**C**) Restored overexpression of FOXO3a in A549 cells with increasing TCRP1, the protein levels of FOXO3a, p21 and p27 were determined by western blot assays. Cell viability assays (**D**) and BrdU assays (**E**) were performed to verify the proliferation ability of the mentioned cells. *vs* TCRP1-KD, *vs* Control, ^**^P < 0.01. (**F**) Flow cytometry assays were employed to measure cell cycle of mentioned cells. *vs* TCRP1-KD, *vs* Control, ^*^P < 0.01.

To further determine the biological role of FOXO3a inhibition in TCRP1-mediated cell-cycle progression, TCRP1-knockdown cells were transiently transfected with FOXO3a shRNA to block the expression of FOXO3a. We found that knockdown FOXO3a reversed the expression of p21 and p27 in TCRP1-knockdown H1299 and H1975 cells (Fig. 7A, B). Consistently, overexpression of FOXO3a also increased the expression of p21 and p27 in TCRP1-overexpressing A549 cells (Fig. 7C). Importantly, the cell growth inhibition triggered by knocking down TCRP1 is completely rescued by FOXO3a shRNA. In contrast, overexpression of FOXO3a significantly reversed TCRP1-induced cell proliferation (Fig. 7D, E). Moreover, cell-cycle analyses indicated that silencing of FOXO3a significantly decreased the G1-phase ratio in TCRP1-knockdown H1299 and H1975 cells (Fig. 7F). Ultimately, these results suggested that TCRP1 promotes NSCLC cell-cycle progression by repressing the expression of FOXO3a.

## Discussion

**Fig. 8:**
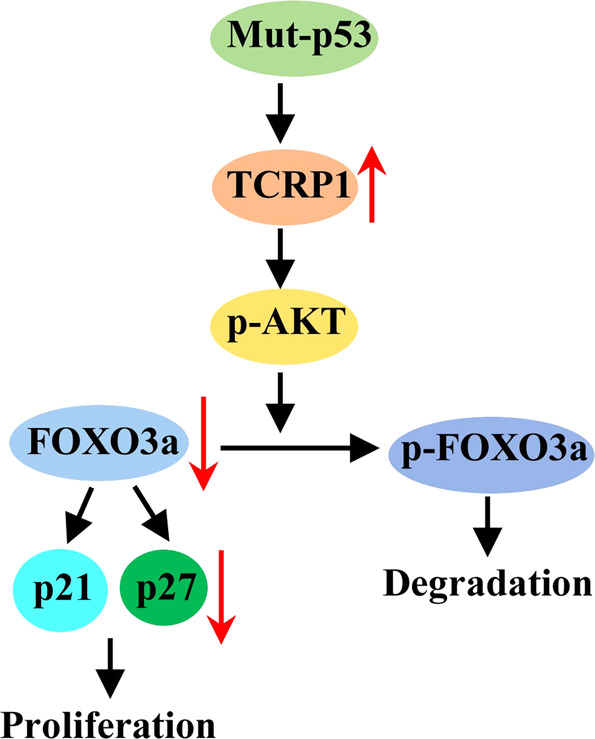
A proposed model of the mechanism by which TCRP1 enhances NSCLC cell proliferation.

In this study, we unveiled that TCRP1 was strongly accelerated in NSCLC via mutant p53. TCRP1 significantly promoted NSCLC cells proliferation and tumor growth in vitro and in vivo. On one hand, TCRP1 interacted with FOXO3a to facilitate its ubiquitination in the cytoplasm. On the other hand, TCRP1 enhanced AKT-mediated phosphorylation and inhibited nuclear localization of FOXO3a, thus regulated cell cycle in NSCLC cells (Fig. 8).

Physiologically, fam168a acted as a novel transcriptional factor in oligodendrocyte differentiation and the subsequent myelin cell development in zebrafish [24]. Besides, many efforts were made to explore the function and mechanism of TCRP1 in cancer progression. Wang et al. suggested that TCRP1 promoted cell transformation and tumorigenesis through hyperphosphorylation of PDK1 and AKT1 in NIH/3T3 cells [22]. The results elucidated that lycorine promoted apoptosis and autophagy through suppressing TCRP1/AKT/mTOR pathway in Hepatocellular carcinoma [25]. It is displayed that FAM168A might act as a linker protein binding to BCR-ABL1 and AKT1, further enhanced the activity of AKT1/NF-κB signaling pathway, thus, increased of G2/M phase cells and Cyclin B1 level in chronic myeloid leukemia [23]. Also, we found TCRP1 was highly expressed in NSCLC with mutant p53. Functional study revealed that TCRP1 promoted cells proliferation and tumor growth via decreasing p21 and p27 in NSCLC. Compelling evidence reported that FOXO3a induced cell cycle arrest and apoptosis via transcriptional regulation of p21 and p27 [26]. And RNA-sequencing assays suggested that TCRP1-regulated genes were significantly enriched in FOXO signaling pathway. Therefore, we carried out experiments to confirm that TCRP1 markedly upregulated phosphorylated FOXO3a levels at Thr32 residues and downregulated FOXO3a protein levels. It is reported that ERK directly interacted with and phosphorylated FOXO3a at Ser 294, Ser 344, and Ser 425, and finally degraded FOXO3a via an MDM2-mediated ubiquitin-proteasome pathway [27]. To verify this, we performed CO-IP assay and found TCRP1 interacted with FOXO3a to encourage its ubiquitination. Several studies have showed that TCRP1 could active AKT [16, 22, 25]. As known, Thr308 and Ser473 were important sites for AKT activation [28]. And FOXO3a was identified as a key target of the PI3K/AKT signaling pathway [29]. Similarly, we found that restoring TCRP1 evidently increased phosphorylation levels of AKT at Ser473 and FOXO3a at Thr32. AKT inhibitor could reverse the effect of TCRP1 on FOXO3a phosphorylation. In the future, we will focus on the mechanism of FOXO3a ubiquitination mediated by TCRP1.

The regulation mechanism of TCRP1 expression pattern causes much more attention. It is exhibited that WNT/NOTCH-hoxb5b signaling axis mediated Cu-induced myelin and axon defects via hypermethylated pou3f1 and fam168a/b24. TCRP1 was identified as a direct functional target of miR-493. miR-493 promoted the chemosensitivity of NSCLC cells to cDDP through impairing the effect of TCRP1 on DNA damage repair and cell apoptosis [30]. Previously, the bioinformatics analysis combing with luciferase reporter assay and ChIP assay discovered that c-Myc could directly bind to TCRP1 promoter to upregulate its expression [31]. It is illustrated that lycorine decreased TCRP1 protein levels through promoting TCRP1 degradation in hepatocellular carcinoma cells [25]. Here, we found 2 putative sites of p53 on TCRP1 promoter. Usually, mutant p53 are unable to coordinate the transcription process contributing to tumor suppression [32]. In fact, p53 mutant proteins were classified as DNA contact mutants (e.g. p53R273H) and conformational mutants (e.g. p53R175H). The former mutation occurred in a DNA binding residues, the latter change caused a loss of wild-type p53 DNA binding [33]. Particularly, mutant p53R175H exclusively upregulated Twist1 to significantly promoted cell migration and invasion in prostate cancer cells [34]. It is demonstrated that wild-type p53 but not p53V143A or p53R175H or p53R280K transcriptionally repressed CXCR4 expression in breast cancer cells [35]. Consistently, we found that wild-type p53 but not mutant p53 (p53V143A or p53R175H) could transcriptionally downregulate TCRP1 through directly binding to TCRP1 promoter. Moreover, the mRNA level of TCRP1 in LUAD tissues with wild-type p53 was sharply lower than that in LUAD tissues with mutant p53 via TCGA data. The similar results were got in NSCLC tissues by IHC assays.

In summary, TCRP1/AKT/FOXO3a axis provides a complementary and more comprehensive understanding of NSCLC and offers an opportunity to translation of basic research to potential treatment in the clinic.

## Supplementary information

supplement figlegend

supplement figure 1

supplement figure 2

## Data Availability

All data needed to evaluate the conclusions are present in the paper. Additional data related to this paper may be requested from the authors.
