# Supplementary material for: TCRP1 activated by mutant p53 promotes NSCLC proliferation via inhibiting FOXO3a
Source: Oncogenesis. 2022 Apr 22;11(1):19. doi: 10.1038/s41389-022-00392-9 (PMC9033812; doi:10.1038/s41389-022-00392-9)
Supplement: Supplementary file 1 — supplement figlegend [file 41389_2022_392_MOESM1_ESM.doc]

Supplement Figure legend

**Fig S1. The effect of restoring and knocking-down p53 in NSCLC cells.** (A) Plasmids of wild type p53 (wt-p53) and mutant p53 (p53R175H and p53V143A) were transfected into H1299 cells, the expression of p53 in mentioned cells were estimated by qRT-PCR and western blot assays. *vs* Con, ^**^ *P*<0.01. (B) ShRNAs targeting p53 and control group were introduced into A549 cells, the expression of p53 in these cells were estimated by qRT-PCR and western blot assays. sh-p53-3# were used in the following study for its best inhibiting effect on p53. *vs* sh-con, ^*^ *P*<0.05, ^**^ *P*<0.01.

**Fig S2. TCRP1 promoted cell proliferation in NSCLC cells.** (A) The clone formation ability of TCRP1 KD cells and control cells were assessed, respectively. *vs* Control, ^**^ *P*<0.01. Lentivirus overexpression plasmid of TCRP1 and control plasmid were transfected into A549 cells, respectively. Western blot assays were used to measure the protein level of TCRP1 in each group (B). (C) Cell viability and BrdU assays were managed to assess the proliferation ability of A549 cells with overexpressing TCRP1 and control cells. *vs* LV-Control, ^**^ *P*<0.01.
