# Supplementary figures and images for: TCRP1 activated by mutant p53 promotes NSCLC proliferation via inhibiting FOXO3a
Source: Oncogenesis. 2022 Apr 22;11(1):19. doi: 10.1038/s41389-022-00392-9 (PMC9033812; doi:10.1038/s41389-022-00392-9)

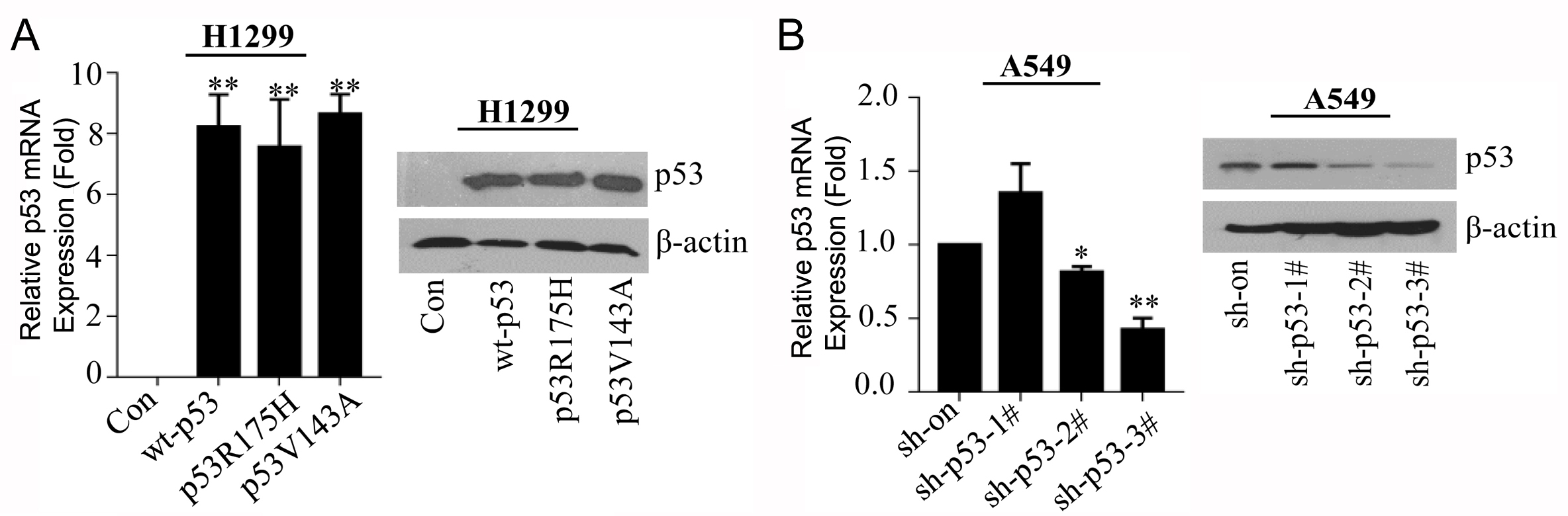

Supplement: Supplementary file 2 — supplement figure 1 [file 41389_2022_392_MOESM2_ESM.jpg]

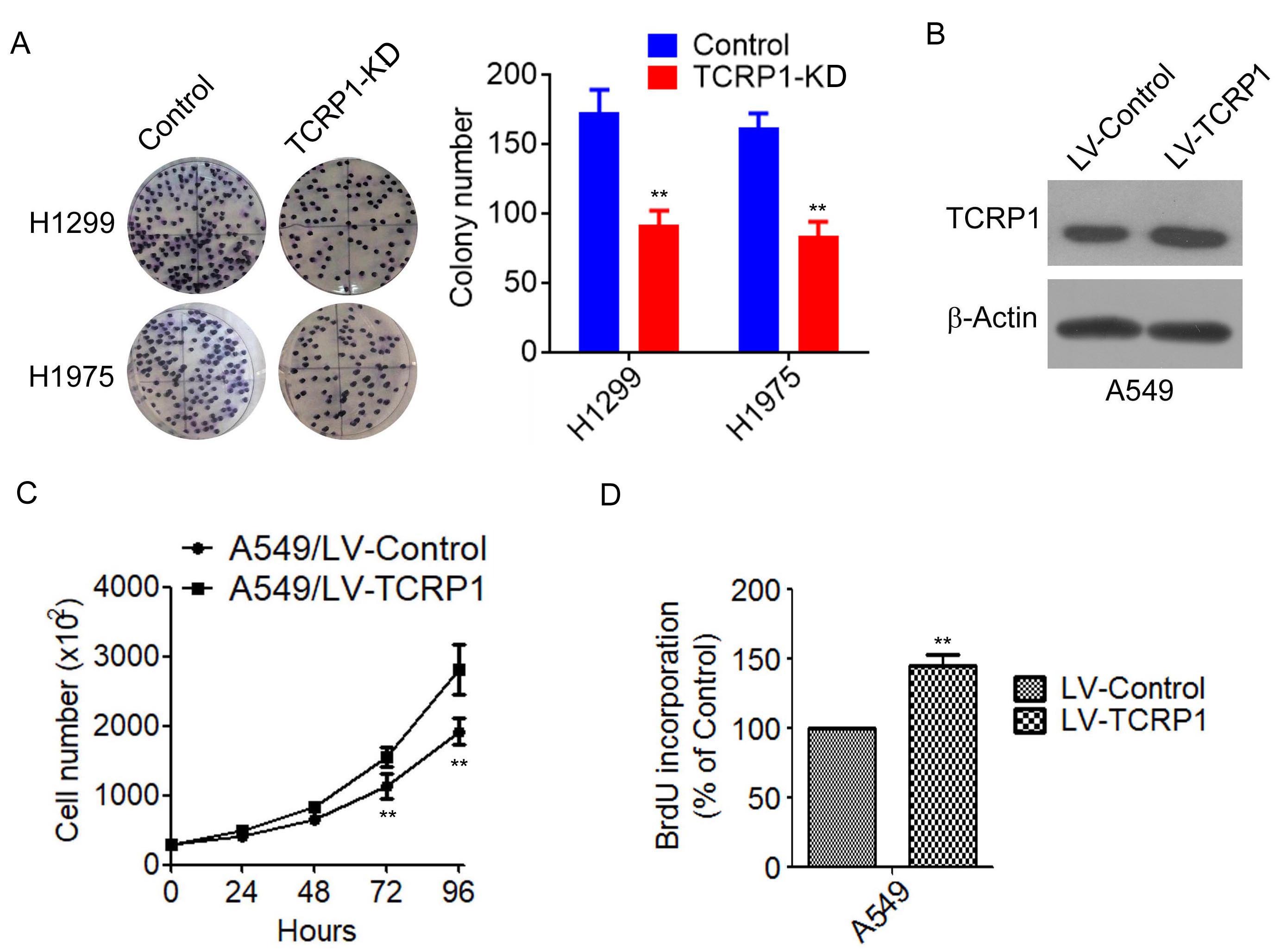

Supplement: Supplementary file 3 — supplement figure 2 [file 41389_2022_392_MOESM3_ESM.jpg]
